# Protocol for the development and validation of a Polypharmacy Assessment Score

**DOI:** 10.1186/s41512-024-00171-7

**Published:** 2024-07-16

**Authors:** Jung Yin Tsang, Matthew Sperrin, Thomas Blakeman, Rupert A. Payne, Darren M. Ashcroft

**Affiliations:** 1 Centre for Primary Care and Health Services Research, School of Health Sciences, University of Manchester, Manchester, M13 9PL UK; 2 NIHR Greater Manchester Patient Safety Research Collaboration (GMPSRC), Faculty of Biology, Medicine and Health, Manchester Academic Health Sciences Centre (MAHSC), University of Manchester, Manchester, UK; 3 Division of Informatics, Imaging and Data Sciences, School of Health Sciences, University of Manchester, Manchester, UK; 4 Department of Health and Community Sciences, University of Exeter Medical School, Exeter, UK; 5 Division of Pharmacy and Optometry, School of Health Sciences, University of Manchester, Manchester, UK

**Keywords:** Prediction modelling, Risk calculator, Polypharmacy, Multimorbidity, Overtreatment, Inappropriate prescribing

## Abstract

**Background:**

An increasing number of people are using multiple medications each day, named polypharmacy. This is driven by an ageing population, increasing multimorbidity, and single disease-focussed guidelines. Medications carry obvious benefits, yet polypharmacy is also linked to adverse consequences including adverse drug events, drug-drug and drug-disease interactions, poor patient experience and wasted resources. Problematic polypharmacy is ‘the prescribing of multiple medicines inappropriately, or where the intended benefits are not realised’. Identifying people with problematic polypharmacy is complex, as multiple medicines can be suitable for people with several chronic conditions requiring more treatment. Hence, polypharmacy is often potentially problematic, rather than always inappropriate, dependent on clinical context and individual benefit vs risk. There is a need to improve how we identify and evaluate these patients by extending beyond simple counts of medicines to include individual factors and long-term conditions.

**Aim:**

To produce a *Polypharmacy Assessment Score* to identify a population with unusual levels of prescribing who may be at risk of potentially problematic polypharmacy.

**Methods:**

Analyses will be performed in three parts:

1. A prediction model will be constructed using observed medications count as the dependent variable, with age, gender and long-term conditions as independent variables. A ‘*Polypharmacy Assessment Score*’ will then be constructed through calculating the differences between the observed and expected count of prescribed medications, thereby highlighting people that have unexpected levels of prescribing.

Parts 2 and 3 will examine different aspects of validity of the *Polypharmacy Assessment Score*:

2. To assess ‘construct validity’, cross-sectional analyses will evaluate high-risk prescribing within populations defined by a range of *Polypharmacy Assessment Scores*, using both explicit (STOPP/START criteria) and implicit (Medication Appropriateness Index) measures of inappropriate prescribing*.*

3. To assess ‘predictive validity’, a retrospective cohort study will explore differences in clinical outcomes (adverse drug reactions, unplanned hospitalisation and all-cause mortality) between differing scores*.*

**Discussion:**

Developing a cross-cutting measure of polypharmacy may allow healthcare professionals to prioritise and risk stratify patients with polypharmacy using unusual levels of prescribing. This would be an improvement from current approaches of either using simple cutoffs or narrow prescribing criteria.

## Introduction

Polypharmacy is broadly defined as the use of multiple medicines [1, 2]. Over a third of people over 65 are taking more than five regular medicines, with almost a quarter taking eight or more [3]. This has an ever escalating prevalence, driven by an ageing population, multimorbidity (multiple long-term conditions) and clinical guidance focussed on individual diseases [2, 4]. Medications carry clear benefits, yet polypharmacy is linked to adverse consequences including poor patient experience, unplanned hospitalisation and death [1, 3]. Adverse reactions and medication errors are directly linked to the number of medicines prescribed, increasing health service utilisation, reducing adherence and decreasing quality of life [5–7]. In England, it is estimated that 10% of medications are inappropriate and potentially harmful, costing the NHS up to £1 billion in medications wastage alone [8]. Problematic polypharmacy has been defined as ‘the prescribing of multiple medications inappropriately, or where the intended benefit of the medication is not realised’ [1].

Better methods to identify and evaluate patients with problematic polypharmacy are crucial [1]. There is no consensus on a definition for polypharmacy, with significant variations in approaches to targeting problematic polypharmacy [8, 9]. The World Health Organization defines polypharmacy as four or more medicines, and academic studies most commonly use five or more, with the NHS national polypharmacy indicators starting at eight or more [8, 10, 11]. However, these simple counts or thresholds ignore individual patient factors and clinical appropriateness [1, 12, 13]. This makes it difficult to define and measure outcomes, with interventions being unable to effectively target the optimal population [1, 13]. Other targeted approaches frequently adopt drug-specific, explicit prescribing criteria such as STOPP/START or the Beers List [13–15]. However, these methods focus on individual examples of high-risk prescribing within a limited number of conditions, rather than polypharmacy as a whole [1, 12]. There have also been numerous attempts to develop prognostic models for adverse drug reactions, yet to date these have demonstrated inadequacies in performance and clinical application, particularly during external validation [16, 17].

Evidence suggests that a comprehensive risk stratification approach is needed to identify and target patients with problematic polypharmacy whilst taking into account individual patient factors [1, 8, 18]. Hence, we propose a novel approach. First, using a regression model, we plan to predict the ‘expected’ count of prescribed medications for each patient, given individual patient characteristics and clinical diagnoses. Then by calculating the discrepancies between the observed and expected count of medications, we can highlight people with unusual levels of prescribing in the context of their clinical and demographic status. This may help prioritise people who are more at risk of problematic polypharmacy. For example, someone on 20 medicines but expected to be taking 5, based on their age and multimorbidity, is likely to require more attention. This protocol describes our approach to the development and validation of a Polypharmacy Assessment Score.

## Aims and objectives

### Aim

To produce a *Polypharmacy Assessment Score* to identify a population with unusual levels of prescribing who may be at risk of potentially problematic polypharmacy.

### Objectives

1. Develop a *Polypharmacy Assessment Score* that accounts for individual patient factors and clinical diagnoses to identify a population with unusual levels of prescribing.
2. Assess the ‘construct validity’ of the score, by estimating the association the *Polypharmacy Assessment Score* with high-risk prescribing.
3. Estimate ‘predictive validity’ of the score, by estimating the risk of adverse outcomes (including adverse drug reactions, hospitalisation and death) within stratified populations of the *Polypharmacy Assessment Score*.

## Methods

**Fig. 1:**
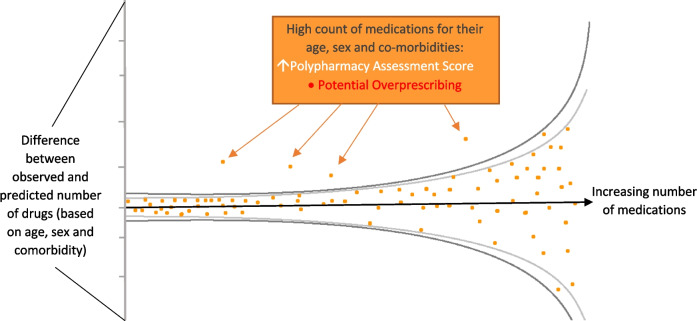
Illustration of the Polypharmacy Assessment Score: a model will determine the predicted count of medications for individual patients given their age, gender and long-term conditions. By then calculating the discrepancy between the observed and predicted number of prescribed medications, the Polypharmacy Assessment Score illustrated by the funnel plot will thus identify those who receive unexpectedly high levels of medicines relative to their multimorbidity

This protocol is guided by the Transparent Reporting of a multivariable prediction model for Individual Prognosis Or Diagnosis (TRIPOD) [19]. However, although our model uses prediction statistics, there are subtle differences compared with the construction of a traditional prediction model. In particular, as defining whether polypharmacy is actually problematic requires implicit clinical judgement, the principal output of the Polypharmacy Assessment Score is to provide a risk stratification approach to identifying unusual levels of prescribing, akin to funnel plot approaches used for audit (Fig. 1) [20]. This approach allows visualisation of the dispersion of outcomes, allowing prioritisation of a group of patients with unexpected levels of prescribing, defined at an acceptable threshold for representativeness and utility. Therefore, clinical representation and appropriateness need to be carefully considered and balanced with predictive performance. For example, deprivation will be excluded, as a person who is more deprived should receive the same medical care as a person who is less deprived if they have the same clinical characteristics. Also, the dependent variable of our model is observed medication count, rather than a clinical outcome for prognostic prediction.

### Data source

The Clinical Practice Research Datalink (CPRD) is a large longitudinal database of general practice electronic healthcare records of over 60 million UK patients. CPRD Aurum data is obtained from the most widely used clinical information system in UK primary care (EMIS®) [21]. It includes detailed coded patient-level data on demographics, clinical events, diagnoses and medicines prescribed. The data is nationally representative in terms of age, gender and ethnicity, collated from over 1700 primary care practices in England [21, 22].

For additional analyses in objectives 2 and 3, additional patient-level linkages will be requested to English national administrative data on dates and diagnoses for hospital admissions (Hospital Episode Statistics, HES), Office for National Statistics (ONS) mortality records, and small area measures of socioeconomic deprivation (Index of Multiple Deprivation, IMD) based on patient residential postcodes.

### Participants

Study participants will be aged 40 years or over. Regular medication exposure will be defined by > 90-day prescription length within 180 days and with at least one issue within the last 3 months prior to index date. Participants must have at least 1 year of continuous practice registration before study entry, thus ensuring reliable measures of medication use and baseline covariates. Participants will only be included if records are defined as acceptable for research purposes by CPRD and eligible for HES, ONS and IMD linkages.

The study period will be between 1st Jan 2000 and 31st Jan 2020. Random index dates for each patient will be utilised to avoid time-sensitive (e.g. seasonal) variations in prescribing, with a sensitivity analysis performed on fixed calendar time index dates and performing landmark analysis to compare results. Study exit will be defined as ended on the earliest of the following: the patient’s death, the date the patient transferred out of their practice, the last date of data collection from the patient’s practice, or the end of the study period.

### Outcome

For objective 1 (prediction model), the primary outcome is the observed count of regular long-term medications. Using this, we can calculate the difference between the observed and predicted count of prescribed medications for individual patients. This difference will represent the *Polypharmacy Assessment Score*, with a large positive difference (in other words, observed greater than predicted) representing a greater level of potential over-prescribing and a large negative difference representing a greater level of potential under-prescribing (Fig. 1).

For objective 2 (construct validity), the primary outcome is high-risk prescribing (using both explicit and implicit criteria).

For objective 3 (predictive validity), the primary outcome is adverse drug reactions, with secondary outcomes being unplanned hospitalisation and all-cause mortality.

### Predictors

Our main predictors include age, gender and long-term conditions. We will include a list of 37 long-term conditions used in CPRD from previous studies and will be guided from by recommendations from the international consensus on definition and measurement multimorbidity [4, 23, 24]. This is due to initial considerations for optimising simplicity, clinical representativeness (e.g. predictors such as deprivation will be intentionally excluded, as explained above) and clinical utility (e.g. other common predictors such as height and weight are likely to have inaccuracies and significant levels of missing data). However, we will explore whether other clinical factors (e.g. smoking, blood pressure) may improve model performance.

### Sample size

The approximate sample size is 8.7 million patients, based on the number of adults aged 40 years and over that are eligible for linkage and limited by random index dates [1]. Our analyses are exploratory, but following current literature and estimations using a global shrinking factor of 0.9, for a hypothetical model with up to 80 predictors, a standard deviation of 2.5 and an adjusted R-squared of 0.7 based on a previous model on multimorbidity [25]; an approximate minimum sample of 577 participants (7.21 events per predictor) is required to precisely estimate calibration and discrimination measures for a continuous outcome [26]. A more conservative estimation, using a lower R-square of 0.5, would require a minimum of 1005 participants (12.6 events per predictor). As such, our large, estimated sample size of 1.5 M participants is sufficient and importantly allows a wide range of different prescribing and patient characteristics to be represented.

### Missing data

We will only include patients with complete data on age and gender; this is expected to exclude a negligible number of patients. The absence of a clinical diagnosis code will be taken as the absence of that condition, and so we will not have missing data on conditions. In UK primary care practice, virtually, all prescriptions are issued electronically; missing prescriptions will therefore be assumed to mean no drug has been issued. Partially incomplete prescription data will be handled similar to previous studies by using a stepwise algorithm to impute any missing quantity, dose and duration taking into account previous prescriptions, concurrent prescriptions and other patient and practice prescriptions [27, 28].

For objectives 2 and 3, ethnicity and IMD are expected to have small proportions of missing data and will be addressed by creating a missing category. Biological measurements (such as height, weight and blood pressure), smoking status and alcohol use are expected to have missing data. The above variables and other patterns of missing data will be examined, and, depending on the proportion of missing data and established assumptions, missing data will be addressed using a combination of multiple imputation techniques (e.g. predictive mean matching (PMM) for our numeric data and polytomous regression imputation for unordered categorical data), creating a missing category or listwise exclusion for variables containing a small proportion of missing data.

### Statistical analysis methods

Analyses will be performed using R software (version 4.3.2) in three parts:

- Objective 1 (prediction model): A prediction model will be constructed using observed medications count as the dependent variable, with age, gender and long-term conditions as independent variables. A *Polypharmacy Assessment Score* will then be constructed through calculating the differences between the observed and predicted count of prescribed medications, thereby highlighting people that have unexpected levels of prescribing. We will perform further exploratory analyses to optimise utility of the score, including presenting the score primarily as a continuous scale (e.g. using absolute vs relative differences in observed and predicted medication count) or using an additional categorical output (e.g. drawing thresholds for high, medium and low scores), and this will be further determined based on an analysis on distribution of scores within the population and input from a multidisciplinary panel of experts.
- Objective 2 (construct validity): Cross-sectional analyses will estimate the prevalence of high-risk prescribing within populations (using both explicit and implicit criteria*)* with a range of different *Polypharmacy Assessment Scores* (e.g. higher, medium and normal scores).
- Objective 3 (predictive validity): A retrospective cohort study will explore differences in clinical outcomes (adverse drug reactions, unplanned hospitalisation and all-cause mortality) between differing scores of the *Polypharmacy Assessment Score*, again compared to standard cutoffs of medication count.

#### Handling of predictor variables

The model will adjust for key predictors including age (continuous variable), gender (binary variable) and multiple long-term conditions (each condition as a binary variable). The model will first derive a ‘weight’ for each specific long-term condition. As a higher number of medications will be expected for certain clusters of long-term conditions (e.g. diabetes, cardiovascular disease and mental health condition), we will then explore whether accounting for prespecified clusters of conditions may improve the model [29, 30]. This will incorporate a range of interaction terms (likely negative, as similar conditions will result in fewer medicines compared to discordant conditions) that need to be included in the model, based on known overlaps in medications for related conditions and a forward selection algorithm to search for further important two-way interactions [31].

#### Type of model

We will use a multivariable Poisson regression model. However, generalised linear frameworks (e.g. quasi-Poisson models), negative binomial regression models and zero-inflated regression models will also be tested for best fit, if there is evidence of overdispersion [32].

#### Predictor selection before modelling

As described above, age, gender and multiple long-term conditions have been selected considering clinical representativeness and utility. However, we will explore whether other clinical factors (e.g. smoking, blood pressure, kidney function) may improve model performance.

#### Predictor selection during modelling

Selection of terms in the optimal model will be determined using the least absolute shrinkage and selection operator (LASSO) [33]. To handle specified interactions, we will utilise hierarchical group-lasso regularisation [34].

#### Model performance

Performance of the model will be assessed using mean squared error (applied to log counts). Calibration will be assessed using calibration plots and estimation of calibration intercept and slopes along with calibration in the large (CITL) [35]. Discrimination will be reported using area under the receiver operating characteristics curves (AUROC) reflecting performance at different thresholds, but is not our primary concern as specified above.

#### Internal and external validation

Objective 1 (prediction model): We will utilise ‘internal–external cross validation’ as the most appropriate technique given our large sample size and clustered dataset at practice level [36, 37]. This is a recognised method using data from all but one practice to estimate the prediction model and then uses the one remaining practice to evaluate the performance of the model. This process is systematically repeated by rotating the omitted practice to produce multiple estimates of model performance. Unlike standard internal validation methods (e.g. cross-validation and bootstrapping) which compares model reproducibility between individuals from the same population, ‘internal–external cross validation’ focusses on comparing reproducibility between clusters (in this case — practices) [36, 37]. Model performance estimates will be combined using random-effect meta-analysis [38]. This will evaluate the accuracy of practice-specific performance estimates and also quantify the heterogeneity in model performance across different practices. If the model performs adequately, further external validation may be subsequently performed on external databases, to demonstrate transportability of the Polypharmacy Assessment Score [38]. Local calibration of the models will still be expected for increased applicability to local contexts during future implementation.

#### Additional analyses for validation

Two further analyses will explore construct and predictive validity of the score:

Objective 2 (construct validity): Cross-sectional analyses will first use logistic regression to examine prevalence of high-risk prescribing (composite outcome of drug-drug and drug-disease interactions), using either STOPP/START criteria (the NICE recommended screening tool) for adults ≥ 65 years or the related PROMPT criteria for adults < 65 years, as proxy measures [39, 40]. These criteria will be initially analysed as the binary presence of each individual explicit potentially high-risk prescribing criteria. The same random index dates will be utilised as above, and each participant will be followed up for 12 months from index date to estimate prevalence. This will compare the positive predictive value of populations with different levels of *Polypharmacy Assessment Score* (e.g. higher, medium and normal scores) to standard cutoffs (defined as simple counts of 5, 10 and 15 or more regular medications). A higher positive predictive value detected from patients with higher scores of the *Polypharmacy Assessment Score* would suggest that it is a better measure of high-risk prescribing than normal scores and simple counts.

In addition, a separate cross-sectional analysis will compare a random sample of 30 patients with high and low *Polypharmacy Assessment Scores *to 30 patients with normal scores (absolute and relative differences evaluated separately) evaluating elements of inappropriate prescribing using the Medication Appropriateness Index, ratified by two independent assessors [41]. This will enable a further comparison of the sensitivity and specificity of *Polypharmacy Assessment Scores* in identifying high-risk prescribing.

Objective 3 (predictive validity): Several clinical outcomes (primarily adverse drug reactions, but also unplanned hospitalisation, and all-cause mortality) will be assessed in a retrospective cohort study using the above random index dates, with sensitivity analyses on further fixed dates to explore seasonal variations), to examine whether the *Polypharmacy Assessment Score* predicts relevant outcomes. Exposure will be defined through different levels of *Polypharmacy Assessment Scores* (e.g. higher vs normal scores). Predictive validity for each outcome will be compared by repeating the cohort study using standard cutoffs (5, 10 or 15 more regular medications) as exposure. Clinical outcomes will be measured at 1- and 5-year follow-up and include adverse drug reactions, unplanned hospitalisation and all-cause mortality using Cox regression. Higher *Polypharmacy Assessment Scores* are anticipated to exhibit acceptable predictive accuracy for clinical outcomes, and better predictive accuracy for adverse drug reactions compared to normal scores and to lower counts of standard cutoffs (e.g. patients on five or more medications). This would serve as further evidence of validity in relation to outcomes.

## Discussion

Compared to current methods, our approach may allow the prioritisation of patients with problematic polypharmacy in a more individualised and holistic manner. Our approach is intentionally pragmatic in adjusting for age, gender and multiple long-term conditions, in order to maximise explainability and implementability, and is designed as a generic measure across medications and conditions for broad applicability. We have further planned qualitative and implementation research with clinical professionals to iteratively explore clinical utility and further develop validity [42].

There are several limitations to consider. Given the complexities of prescribing decisions, the *Polypharmacy Assessment Score *will not perfectly identify every individual patient with high-risk prescribing or replace clinical judgement. Some of these complexities will not be captured in routine data, such as the doctor-patient relationship, changing guidelines and differing opinions from specialists [43]. Although primary care electronic prescription data is reliable, we may miss out exclusively secondary care prescriptions and over-the-counter medications. Whilst not our primary focus, there is also some potential to highlight patients with underprescribing. Appropriateness is not explicitly measured by our score and inherently requires a consultation and shared decision between clinicians and patients [1, 8]. Hence, the score only highlights ‘potentially’ problematic polypharmacy. Therefore, the added value of this score is to prioritise a group of patients with unexpected levels of prescribing given their individual characteristics and multimorbidity, who we hypothesise to be at higher risk of problematic polypharmacy.

## Data Availability

In this study, we will use anonymised patient-level data from the CPRD that are not publicly available due to confidentiality considerations. However, researchers can access CPRD’s databases by contacting the CPRD. Details of the application process and conditions of access are available at https://www.cprd.com/data-access.

## Abbreviations

AUROC: Area under the receiver operating characteristics curve
CITL: Calibration-in-the-large
CPRD: Clinical Practice Research Datalink
HES: Hospital Episode Statistics
IMD: Index of Multiple Deprivation
LASSO: Least absolute shrinkage and selection operator
NHS: National Health Service
ONS: Office for National Statistics
TRIPOD: Transparent Reporting of a multivariable prediction model for Individual Prognosis Or Diagnosis

## References

[1] Duerden M, Avery T, Payne R (2013). Polypharmacy and medicines optimisation.

[2] Guthrie B, Makubate B, Hernandez-Santiago V (2015). The rising tide of polypharmacy and drug-drug interactions: population database analysis 1995–2010. BMC Med.

[3] Hajjar ER, Cafiero AC, Hanlon JT (2007). Polypharmacy in elderly patients. Am J Geriatr Pharmacother.

[4] Barnett K, Mercer SW, Norbury M (2012). Epidemiology of multimorbidity and implications for health care, research, and medical education: a cross-sectional study. The Lancet.

[5] Avery AA, Barber N, Ghaleb M (2012). Investigating the prevalence and causes of prescribing errors in general practice: the PRACtICe study.

[6] Gellad WF, Grenard JL, Marcum ZA (2011). A systematic review of barriers to medication adherence in the elderly: looking beyond cost and regimen complexity. Am J Geriatr Pharmacother.

[7] Nordin Olsson I, Runnamo R, Engfeldt P (2011). Medication quality and quality of life in the elderly, a cohort study. Health Qual Life Outcomes.

[8] The Department of Health and Social Care (2021). National overprescribing review report: good for you, good for us, good for everybody.

[9] Taghy N, Cambon L, Cohen J-M (2020). Failure to reach a consensus in polypharmacy definition: an obstacle to measuring risks and impacts—results of a literature review. Ther Clin Risk Manag.

[10] World Health Organization (2017). Medication without harm: World Health Organization.

[11] NHS Business Services Authority (2017). Medicines Optimisation Polypharmacy Prescribing Comparators.

[12] Gnjidic D, Tinetti M, Allore HG (2017). Assessing medication burden and polypharmacy: finding the perfect measure. Expert Rev Clin Pharmacol.

[13] Rankin A, Cadogan CA, Patterson SM, et al. Interventions to improve the appropriate use of polypharmacy for older people. Cochrane Database Syst Rev. 2018;2018(9). 10.1002/14651858.CD008165.pub4. 10.1002/14651858.CD008165.pub4 PMC6513645 30175841

[14] Cooper JA, Cadogan CA, Patterson SM (2015). Interventions to improve the appropriate use of polypharmacy in older people: a Cochrane systematic review. BMJ Open.

[15] Alldred DP, Kennedy M-C, Hughes C, et al. Interventions to optimise prescribing for older people in care homes. Cochrane Database Syst Rev. 2016;2016(2). 10.1002/14651858.CD009095.pub3. 10.1002/14651858.CD009095.pub3 PMC7111425 26866421

[16] Stevenson J, Williams JL, Burnham TG, et al. Predicting adverse drug reactions in older adults; a systematic review of the risk prediction models. Clin Interv Aging. 2014:1581. 10.2147/CIA.S65475. 10.2147/CIA.S65475 PMC4178502 25278750

[17] Falconer N, Barras M, Cottrell N (2018). Systematic review of predictive risk models for adverse drug events in hospitalized patients: predictive risk models for adverse drug events in hospitalized patients. Br J Clin Pharmacol.

[18] Payne RA, Abel GA, Avery AJ (2014). Is polypharmacy always hazardous? A retrospective cohort analysis using linked electronic health records from primary and secondary care: polypharmacy and hospitalization. Br J Clin Pharmacol.

[19] Collins GS, Reitsma JB, Altman DG (2015). Transparent reporting of a multivariable prediction model for individual prognosis or diagnosis (TRIPOD): the TRIPOD Statement. BMC Med.

[20] Spiegelhalter DJ (2005). Funnel plots for comparing institutional performance. Stat Med.

[21] Herrett E, Gallagher AM, Bhaskaran K (2015). Data Resource Profile: Clinical Practice Research Datalink (CPRD). Int J Epidemiol.

[22] Wolf A, Dedman D, Campbell J (2019). Data resource profile: Clinical Practice Research Datalink (CPRD) Aurum. Int J Epidemiol.

[23] Cassell A, Edwards D, Harshfield A (2018). The epidemiology of multimorbidity in primary care: a retrospective cohort study. Br J Gen Pract.

[24] Ho ISS, Amaya A-L, Ashley A (2022). Measuring multimorbidity in research: Delphi consensus study. BMJ Medicine.

[25] Payne RA, Mendonca SC, Elliott MN (2020). Development and validation of the Cambridge Multimorbidity Score. CMAJ.

[26] Riley RD, Snell KIE, Ensor J (2019). Minimum sample size for developing a multivariable prediction model: part I–continuous outcomes. Stat Med.

[27] Pye SR, Sheppard T, Joseph RM (2018). Assumptions made when preparing drug exposure data for analysis have an impact on results: an unreported step in pharmacoepidemiology studies. Pharmacoepidemiol Drug Saf.

[28] Denholm R, Morris R, Payne R (2019). Polypharmacy patterns in the last year of life in patients with dementia. Eur J Clin Pharmacol.

[29] Forslund T, Carlsson AC, Ljunggren G (2021). Patterns of multimorbidity and pharmacotherapy: a total population cross-sectional study. Fam Pract.

[30] Zhu Y, Edwards D, Mant J (2020). Characteristics, service use and mortality of clusters of multimorbid patients in England: a population-based study. BMC Med.

[31] Leung TI, Dumontier M (2016). Overlap in drug-disease associations between clinical practice guidelines and drug structured product label indications. J Biomed Semantics.

[32] Ver Hoef JM, Boveng PL (2007). Quasi-Poisson vs. negative binomial regression: how should we model overdispersed count data?. Ecology.

[33] Pavlou M, Ambler G, Seaman S (2016). Review and evaluation of penalised regression methods for risk prediction in low-dimensional data with few events. Stat Med.

[34] Lim M, Hastie T (2015). Learning interactions via hierarchical group-lasso regularization. J Comput Graph Stat.

[35] Steyerberg EW (2009). Applications of prediction models.

[36] Takada T, Nijman S, Denaxas S (2021). Internal-external cross-validation helped to evaluate the generalizability of prediction models in large clustered datasets. J Clin Epidemiol.

[37] Debray TP, Vergouwe Y, Koffijberg H (2015). A new framework to enhance the interpretation of external validation studies of clinical prediction models. J Clin Epidemiol.

[38] Debray TP, Moons KG, Ahmed I (2013). A framework for developing, implementing, and evaluating clinical prediction models in an individual participant data meta-analysis. Stat Med.

[39] O’Mahony D, Cherubini A, Guiteras AR (2023). STOPP/START criteria for potentially inappropriate prescribing in older people: version 3. Eur Geriatr Med.

[40] Cooper JA, Moriarty F, Ryan C (2016). Potentially inappropriate prescribing in two populations with differing socio-economic profiles: a cross-sectional database study using the PROMPT criteria. Eur J Clin Pharmacol.

[41] Hanlon JT, Schmader KE (2013). The medication appropriateness index at 20: where it started, where it has been, and where it may be going. Drugs Aging.

[42] Tsang JY, Blakeman T, Sperrin M, et al. Identifying, understanding and addressing problematic polypharmacy within multimorbidity in primary care: National Institute for Health and Care Research (NIHR; 2022 [Available from: https://fundingawards.nihr.ac.uk/award/NIHR302624. Accessed 1 May 2023.

[43] Spinewine A, Schmader KE, Barber N (2007). Appropriate prescribing in elderly people: how well can it be measured and optimised?. The Lancet.

